# Kaplan Lesion in a 10‐Year‐Old Boy: A Case Report

**DOI:** 10.1002/ccr3.71168

**Published:** 2025-10-06

**Authors:** Kushal Khanal, Sunil Chaudhary, Shirish Adhikari, Prawesh Singh Bhandari, Nitish Bikram Deo, Suresh Uprety, Sangit Chhantyal

**Affiliations:** ^1^ Department of Orthopedics and Traumatology Institute of Medicine, Maharajgunj Kathmandu Nepal; ^2^ Maharajgunj Medical Campus, Tribhuvan University, Institute of Medicine Kathmandu Nepal

**Keywords:** case report, complex dislocation, dorsal approach, metacarpophalangeal joint

## Abstract

Kaplan's lesion, a rare complex metacarpophalangeal (MCP) joint dislocation in children, is often requires surgical intervention. The dorsal approach minimizes neurovascular risk, while the volar approach allows volar plate repair. A 10‐year‐old boy presented with a left index finger MCP dislocation after trauma. Closed reduction failed, and open reduction via a dorsal approach was performed. Intraoperative findings included volar plate interposition and cartilage fracture. Postoperative immobilization and physiotherapy restored full joint motion at six months. Kaplan's lesion is rare in children, requiring careful management to avoid complications like stiffness or growth arrest. The dorsal approach offers effective visualization and safety. Timely surgical intervention ensures successful outcomes in complex MCP dislocations in children.

**Summary**

- Kaplan's lesion is a rare complex MCP joint dislocation that often requires surgical intervention. The dorsal surgical approach minimizes neurovascular risk, while the volar approach allows direct volar plate repair but has higher complications.
- Pediatric cases require special attention to detect cartilage fractures and avoid growth arrest, emphasizing the importance of early recognition and proper management.
- Prompt reduction, appropriate immobilization, and early mobilization are key to minimizing future complications.
- This case describes the successful management of a rare pediatric Kaplan's lesion using a dorsal surgical approach.

## 1 Introduction

Kaplan's lesion is a complex metacarpophalangeal (MCP) joint dislocation, which is uncommon in children and is rarely reported [1]. MCP joint dislocations that can be reduced using closed techniques are known as simple dislocations, while those that are irreducible to closed maneuvers and require surgical intervention are known as complex dislocations [2].

Two different surgical approaches are described: the volar and dorsal approaches. The dorsal approach has the advantage of minimal risk to neurovascular bundles compared to the volar approach [3]. While proponents of the volar approach advocate that this approach provides better viewing of the joint and, if needed, repair of the volar plate can be done simultaneously [4].

In this report, we present the case of a 10‐year‐old patient who sustained a complex index finger MCP joint dislocation and was successfully managed in a tertiary care setting. This case has been reported as per SCARE 2023 guidelines [5].

## 2 Case History/Examination

**FIGURE 1:**
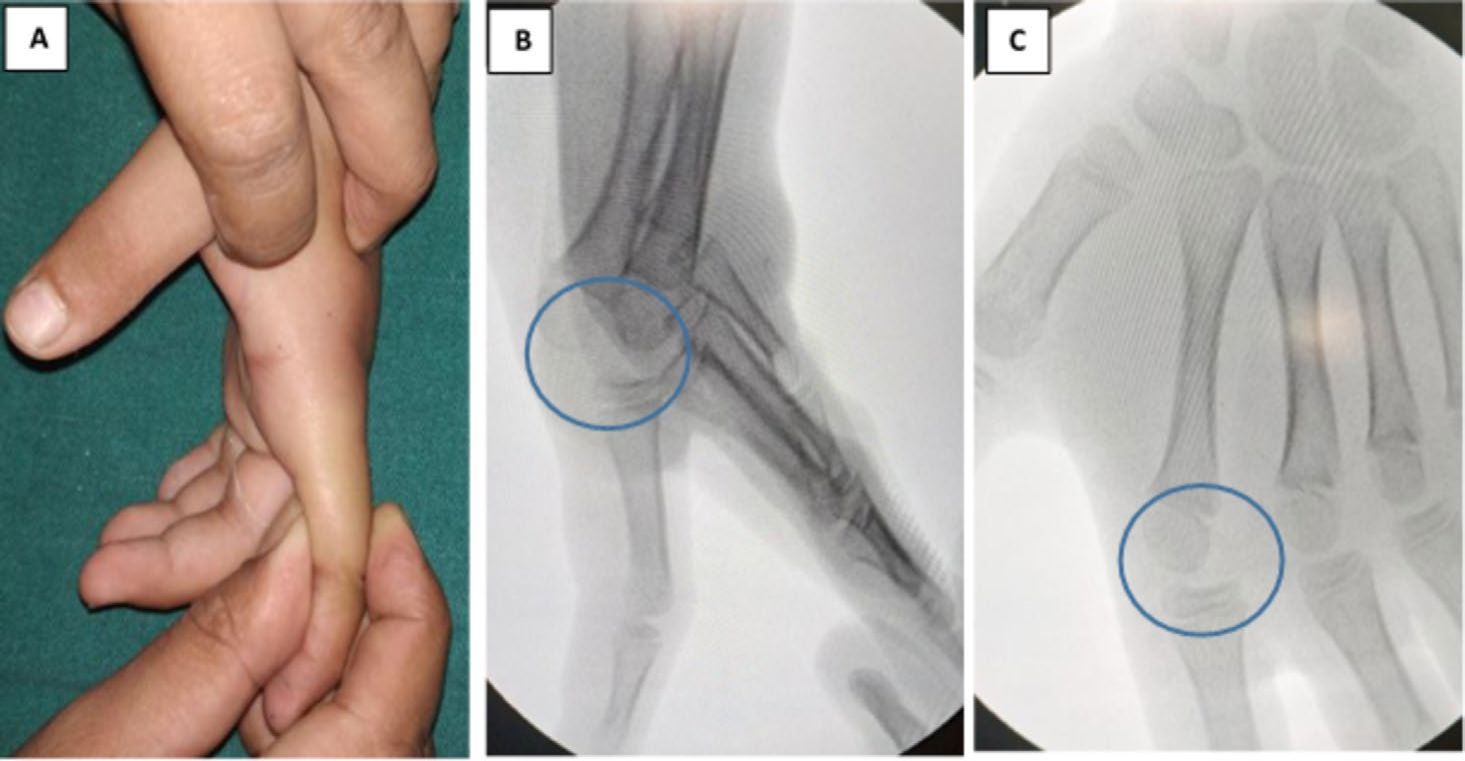
Pre‐operative clinical image (A) and radiographs (B, C) showing left hand index finger MCP joint dislocation (rings).

A 10‐year‐old male presented to the Emergency Room (ER) with complaints of pain and swelling over the left index finger following a forceful impact on the wooden part of the bed with the palmar aspect of the left hand 2 days back. On examination, there was mild hyperextension deformity, along with swelling and tenderness over the left second MCP joint (Figure 1A). Movement was also painfully restricted.

## 3 Methods

X‐ray radiographs revealed a left index finger MCP dislocation (Figure 1B,C). After multiple unsuccessful close reduction attempts, the patient was managed with open reduction. Under general anesthesia, the second MCP joint was approached dorsally with a curvilinear incision. The sagittal band and underlying capsule were split longitudinally to expose the volar plate, which was interposed between the head of the metacarpal and the base of the proximal phalanx. In addition, there was an articular cartilage fracture. The intraoperative diagnosis was “Complex Left index finger MCP dislocation” also known as a Kaplan lesion. MCP joint reduction was achieved. A below‐elbow dorsal slab with MCP in 90‐degree flexion was applied.

Postoperative radiographs were taken to look for the adequacy of reduction.

## 4 Conclusion and Results

The patient had no postoperative complications. He was discharged on the fourth day after surgery with pain medications. We advised the patient to maintain the slab for 3 weeks followed by active range of motion exercises thereafter.

**FIGURE 2:**
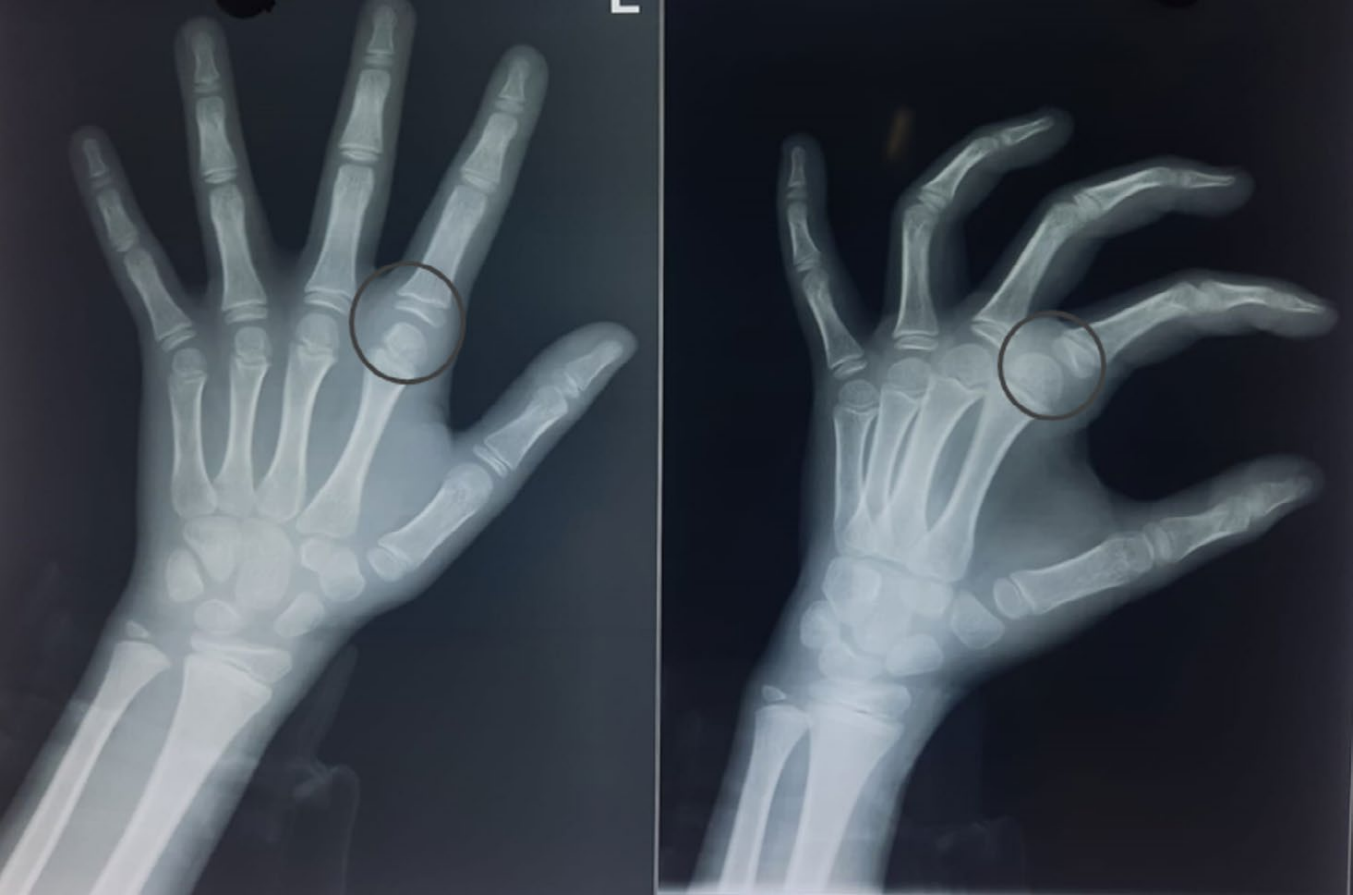
Follow up radiographs at 6 months showing well‐reduced left index finger MCP joint (rings).

At 6 months follow‐up, the patient had regained full range of motion of the index finger MCP joint. Radiographs taken during the same time showed a well‐reduced MCP joint of the left index finger (Figure 2).

## 5 Discussion

MCP joint dislocations are relatively uncommon and occur less often than interphalangeal joint dislocations. An Indian study reported 20 cases in a period of 7 years in 2018. Kaplan's lesion is a rare injury. So far, this is the second case from Nepal that is similar to the first case reported in 2022 [1].

The MCP joint is a synovial condyloid joint that involves motion in multiple planes. The complex shape of the metacarpal head, in addition to the articular surface and supporting soft tissue structures, allows for flexion, extension, adduction, and circumduction. The MCP joint is enclosed in a joint capsule that extends from the metacarpal neck to the base of the proximal phalanx. The joint is reinforced by ligamentous structures on all sides [2]. The primary stabilizer of the MCP joint in adduction and abduction is the radial and ulnar proper collateral ligaments, which originate from the condyles of the metacarpal head to the base of the proximal phalanx and distend obliquely from dorsal proximal to ventral distal direction. This acts as a restraint in MCP flexion. The accessory collateral ligament originates anterior to this ligament and inserts over the volar plate, stabilizing the volar plate. This acts as a static restraint in MCP extension [3]. The volar plate is a fibrocartilaginous structure located at the palmar aspect of the MCP joint. The volar plate is a rectangular reinforcement of the capsule composed of a thick fibrocartilaginous portion distally and a looser membranous portion proximally [2, 4]. Kaplan's original description clearly points out the pathoanatomy of MCP joint dislocation—the fibrocartilaginous plate avulses from its weakest attachment, that is, the volar aspect of the metacarpal neck, with the flexor tendons and the pretendinous band displaced ulnarly and the lumbricals displaced radially to the metacarpal head, trapping the volar plate between the base of the proximal phalanx and the dorsal metacarpal head [1]. They are typically caused by the forced hyperextension of a finger on an extended hand. These structures will form a noose‐like structure around the metacarpal neck as they tighten, preventing closed reduction [6]. The dorsal part of the MCP joint is weakest due to the thin and loose joint capsule dorsally. This predisposes the MCP joint to dorsal dislocations to axial loads after a fall on an outstretched hand due to forceful hyperextension [3]. In complex dorsal MCP joint dislocations, the volar plate has been identified as the most significant barrier to reduction [7, 8]. In addition, the metacarpal head may penetrate through the volar soft tissues and become buttonholed [2, 7]. Complex dislocations occur most often within the index finger. The border digit of the index finger is prone to dislocation because of increased vulnerability to trauma and lack of stabilization by two adjacent deep transverse metacarpal ligaments [2, 8]. Simple dislocations may be reduced by reducing tension on the flexor tendons and applying dorsal pressure to the base of the proximal phalanx. However, distracting the joint may sometimes entrap adjacent structures and convert the dislocation into a complex one because traction on the joint may draw dorsally and completely fold the volar plate into the joint [2, 4, 7, 8]. In the pediatric population, care must be taken due to the possibility of cartilage fractures and growth arrest. Children also present more lax ligamentous structures, making them more susceptible to dislocations [7]. Complex pediatric MCP joint dislocations occur in a similar fashion as those in adults, with differences existing in the absence of sesamoid bone formation and the potential for cartilage fractures on the metacarpal head at the collateral ligament insertions [2].

The clinical examination helps in diagnosing the type of dislocation [4]. In dorsal dislocations, there is puckering of palmar skin due to stretching of the pretendinous and transverse group of fibers of palmar fascia and skin, which are displaced by the metacarpal head. The presence of this sign contraindicates closed reduction [3].

In 1975, Becton et al. described a dorsal open reduction approach with advantages like adequate volar plate visualization with avoidance of any digital neurovascular damage and proper reduction of osteochondral fracture of the metacarpal head [3]. The dorsal approach provides excellent exposure of the volar plate, as well as access to the osteochondral fragments of the metacarpal head. The main disadvantage of the dorsal approach, however, is the longitudinal splitting of the volar plate to reduce the MCP joint, which is irreparable. On the other hand, the advantage of the volar approach is that it allows direct access to the lesion and repair of the volar plate, decreasing the risk of subsequent instability. However, there is a higher risk of neurovascular damage in the volar approach [6, 7, 9]. Barry et al. recommended the volar approach for experienced hand surgeons as it allows for a complete anatomic restoration of the joint to be achieved, but they recommended the dorsal approach for the infrequent hand surgeon as it is a simple, safe choice with apparently stable results [2].

Boden et al. described a percutaneous technique using a skin hook to reduce a complex MCP joint dislocation of the index finger of an 8‐year‐old female. Sodha et al. described another percutaneous technique using a dorsal incision for the reduction of complex MCP joint dislocations in four patients, all performed under local anesthesia. The technique has the advantage of limited risk to neurovascular structures. It also avoids the risks associated with open surgery [2, 9, 10]. Kodama et al. described a case of arthroscopic reduction of the index finger in an 11‐year‐old male. It may damage structures less than open surgery but has the disadvantage of limited visualization of extra‐articular pathology [2].

A lateral approach has also been described by Pereira et al. allowing access to both dorsal and palmar structures. However, the risk of neurological injury is higher than with other approaches, which therefore limits use [7].

Complications associated with dorsal MCP joint dislocations are due to delayed presentation, associated fractures, and prolonged immobilization [4]. Complications of general MCP joint injury include degenerative arthritis from repeated closed reduction attempts, traumatic open reduction or prolonged dislocation, osteonecrosis of the metacarpal head, and stiffness [6]. In children, a unique complication is growth arrest that may occur as a consequence of vascular damage and lead to limited joint motion, a shortened metacarpal, or deformity of the metacarpal head [2].

Following surgical reduction, the joint is immobilized with splints for 2–3 weeks. Early range of motion exercises are to be encouraged after removal of the splint, because a longer duration of splinting will lead to a loss in regaining range of motion [4, 6].

## 6 Conclusion

Early recognition and management of MCP joint dislocations are essential to prevent long‐term functional impairments. Regardless of the chosen approach, prompt joint reduction is crucial to minimize complications and ensure optimal outcomes.

## Author Contributions

**Kushal Khanal:** conceptualization, resources, writing – original draft, writing – review and editing. **Sunil Chaudhary:** conceptualization, data curation, project administration, resources, supervision, visualization. **Shirish Adhikari:** conceptualization, data curation, project administration, resources, supervision, validation. **Prawesh Singh Bhandari:** conceptualization, project administration, resources, supervision, validation. **Nitish Bikram Deo:** conceptualization, data curation, supervision, validation, writing – review and editing. **Suresh Uprety:** conceptualization, resources, supervision, validation. **Sangit Chhantyal:** formal analysis, resources, writing – original draft, writing – review and editing.

## Consent

Written informed consent was obtained from the parents of the patient for publication of this case report and any accompanying images. A copy of the written consent is available for review by the Editor‐in‐Chief of this journal on request.

## Conflicts of Interest

The authors declare no conflicts of interest.

## Data Availability

Data available on request due to privacy/ethical restrictions.

## References

[1] A. K. Thakur , N. M. S. Pradhan , P. Devkota , B. Gyawali , and P. M. Pokhrel , “Kaplan's Lesion in a Child: A Case Report,” JNMA; Journal of the Nepal Medical Association 60, no. 255 (2022): 975–977, 10.31729/jnma.7901. 36705167 PMC9795102

[2] G. Sumarriva , B. Cook , G. Godoy , and S. Waldron , “Pediatric Complex Metacarpophalangeal Joint Dislocation of the Index Finger,” Ochsner Journal 18, no. 4 (2018): 398–401, 10.31486/toj.18.0032. 30559627 PMC6292477

[3] N. P. Mahajan , C. T. Patil , and S. Sangma , “Management of Complex Kaplan's Dislocation by Open Dorsal Approach—A Case Report,” Journal of Orthopaedic Case Reports 11, no. 11 (2021): 84–87, 10.13107/jocr.2021.v11.i11.2526. PMC8930320 35415108

[4] S. Srivastava and S. F. Afaque , “Complex Metacarpophalangeal Joint Dislocation of the Index Finger in Pediatric Age: A Case Report and Review of Literature,” Journal of Orthopaedic Case Reports 12, no. 8 (2022): 102–105, 10.13107/jocr.2022.v12.i08.2984. PMC9831231 36687477

[5] C. Sohrabi , G. Mathew , N. Maria , A. Kerwan , T. Franchi , and R. A. Agha , “The SCARE 2023 Guideline: Updating Consensus Surgical CAse REport (SCARE) Guidelines,” International Journal of Surgery 109, no. 5 (2023): 1136–1140, 10.1097/JS9.0000000000000373. 37013953 PMC10389401

[6] R. R. Patluri and A. Gummadi , “Volar Dislocation of Second Metacarpophalangeal Joint‐Open Reduction With Volar Approach—A Rare Case Report,” Journal of Orthopaedic Case Reports 12, no. 10 (2022): 70–73, 10.13107/jocr.2022.v12.i10.3372. PMC9983376 36874896

[7] Y. Durand , N. N. Rodriguez , S. Georgopoulos , C. Mesoraca , and M. Jaën , “Complex Metacarpophalangeal Joint Dislocation (Kaplan's Lesion) of the Index Finger in a 5‐Year‐Old Patient: A Case Report,” Journal of Orthopaedic Case Reports 14, no. 1 (2024): 141–145, 10.13107/jocr.2024.v14.i01.4178. PMC10823807 38292100

[8] J. M. Pereira , M. Quesado , M. Silva , J. D. Dores Carvalho , H. Nogueira , and J. Alves , “The Lateral Approach in the Surgical Treatment of a Complex Dorsal Metacarpophalangeal Joint Dislocation of the Index Finger,” Case Reports in Orthopedics 2019 (2019): 1063829, 10.1155/2019/1063829. 31093396 PMC6481117

[9] A. Kodama , Y. Itotani , and T. Mizuseki , “Arthroscopic Reduction of Complex Dorsal Metacarpophalangeal Dislocation of Index Finger,” Arthroscopy Techniques 3, no. 2 (2014): e261–e264, 10.1016/j.eats.2013.11.008. 24904773 PMC4044501

[10] S. Sodha , G. D. Breslow , and B. Chang , “Percutaneous Technique for Reduction of Complex Metacarpophalangeal Dislocations,” Annals of Plastic Surgery 52, no. 6 (2004): 562–565; discussion 566, 10.1097/01.sap.0000123351.12922.f4. 15166981

